# Finding successful strategies in a complex urban sustainability game

**DOI:** 10.1038/s41598-021-95199-w

**Published:** 2021-08-03

**Authors:** Bernardo Monechi, Enrico Ubaldi, Pietro Gravino, Ilan Chabay, Vittorio Loreto

**Affiliations:** 1 Sony Computer Science Laboratories, 6, Rue Amyot, 75005 Paris, France; 2 Institute for Advanced Sustainability Studies, Berliner Strasse 130, 14467 Potsdam, Germany; 3 Sapienza University of Rome, Physics Department, Piazzale Aldo Moro 2, 00185 Rome, Italy; 4 Complexity Science Hub Vienna, Josefstädter Strasse 39, 1080 Vienna, Austria

**Keywords:** Sustainability, Psychology and behaviour

## Abstract

The adverse effects of unsustainable behaviors on human society are leading to an increasingly urgent and critical need to change policies and practices worldwide. This requires that citizens become informed and engaged in participatory governance and measures leading to sustainable futures. Citizens’ understanding of the inherent complexity of sustainable systems is a necessary (though generally not sufficient) ingredient for them to understand controversial public policies and maintain the core principles of democratic societies. In this work, we present a novel, open-ended experiment where individuals had the opportunity to solve model urban sustainability problems in a purposeful game. Participants were challenged to interact with familiar LEGO blocks representing elements in a complex generative urban economic indicators model. Players seeks to find a specific urban configuration satisfying particular sustainability requirements. We show that, despite the intrinsic complexity and non-linearity of the problems, participants’ ability to make counter-intuitive actions helps them find suitable solutions. Moreover, we show that through successive iterations of the experiment, participants can overcome the difficulties linked to non-linearity and increase the probability of finding the correct solution to the problem. We contend that this kind of *what-if* platforms could have a crucial role in future approaches to sustainable developments goals.

## Introduction

Our era is often labeled as the Anthropocene due to the scale of modern civilization’s impact on the global environment and all aspects of human societies. Finding and designing pathways to sustainable futures of our societies and economies is of urgent and paramount importance, and so is the promotion of citizens involvement in the governance process. However, the core principle of citizens’ participation in the democratic societies requires that they understand the inherent complexity of sustainable systems. The acknowledgment of these needs led the United Nations to compile in 2015 a list of 17 *Sustainable Development Goals* (SDG)^1^ ideally all nations in the world should achieve by 2030. These goals range from ending world poverty to improving infrastructures, from the promotion of gender equality to increasing access to water and socio-ecological-economic opportunities.

A key SDG challenge is how to govern the increasing rate of urbanization and its social and ecological consequences. SDG 11 focuses on improving living conditions in densely inhabited human settlements, by making them more inclusive, sustainable, connected with rural areas and resilient to shocks^1^. Cities are one of the paradigms of Complex Systems^2^, being made by multi-layered^3^ and highly-interconnected components^4^, and being described generally as out-of-equilibrium systems^5^. The interconnections between cities’ elements are inherently non-linear at every scale, starting from the interactions between their microscopic components^6,7^ to the mutual-influence between their macroscopic properties^4,8^.

Therefore, understanding the intrinsic complex and non-linear mechanisms underlying urban systems is of utmost importance for decision-makers^9^. Serious Games^10^ and Games With A Purpose^11^ represent interesting approaches that allow to build skills and improve understanding of decision-making in complex settings. These games, in fact, are built so to promote players’ learning and behavioral changes, and have been applied to a wide variety problems, e.g., air pollution^12^, noise pollution^13^, energy^14^ and urban networks^15,16^. The importance of serious games has grown in recent years, as witnessed by the many efforts devoted to finding a general framework to validate their effectiveness^17–19^.

In this work, we contribute to the framework of Serious Game applied to urban settings by proposing a novel open-ended playful experiment^20^. In our experiment 7585 voluntary participants interacted with a data-driven mathematical model of urban indicators, trying to understand its functioning and learning to deal with its non-linearity and complexity. Participants were not aware of the model functioning, but interacted with it by building a city made of LEGO bricks, where buildings of different colours represented different functional parts (e.g., orange buildings = housing, red buildings = workplaces). A depth-sensing camera monitored the emerging town above the playing area, recording in this way the number of bricks for each different color code in a continuous manner, thus functioning in analogy to observation by earth-imaging satellites. These estimates represented socio-economic inputs for the data-driven mathematical model, trained on real urban data^4^, that runs underneath. The model encoded effective non-linear interactions between the sensors’ input, and several socio-economic indicators (e.g. employment, produced garbage) returned as feedback to the participants through suitably located displays. Those socio-economic indicators depicted the city’s status and provided the participants with a quick overview of how well the city is functioning and its existing criticalities.

In response to the indicators, participants had the opportunity to modify their city to improve it, either in general terms or accepting specific missions, e.g., reducing unemployment below a given value, increase the average salaries, etc. The game dynamics was such that players had several attempts to fulfil the assigned mission in a given amount of time. Specifically, in each “match” between player and computer, the system provided a randomly generated mission to be accomplished in a given amount of time, typically 5 min.

The installation automatically-collected players’ actions, following the concept of *Stealth Assessment* so that players were engaged in the experiment for the pleasure of the activity itself, removing the idea of being part of an experimental setting^21^. No personal information on the players was ever recorded, and we only logged their game strategies.

Through the collected data, we define a set of features that allows us to discriminate with good accuracy whether the player accomplished the given mission or not. We find that the non-linear interactions among different indicators in the underlying mathematical model significantly affect the success probability. The strength of this non-linearity is not uniform across the space of all the possible city configurations. Depending on the specific area of the configuration space where the player is working, she/he might receive either linear or non-linear responses to the move. In general, starting from regions of the space where interactions are strongly non-linear, and moving towards areas with more linear interactions enhances the success probability. However, we show that players can easily overcome this issue by iterating the match, as the metrics related to non-linearity become irrelevant for predicting the outcome after the first match.

Through the mathematical model accompanying the experiment, we defined an intrinsic metric of *complexity*, characterizing the difficulty of completing each specific mission. High values of this metric correlate with lower success probabilities. Exploiting surveys made either with Complex Systems experts or the general public, we were able to identify counter-intuitive moves, that we called *paradox moves*. These moves challenge the common beliefs about the problem though still aligned with the way the model works. We showed that performing such moves enhanced the probability of a positive outcome. Finally, we observed that matches with the right balance of duration and time spent per move were the most likely successful. In other words, we observed the highest-probability of success when the player performed a few well-considered actions, rather than many rapid trial-and-error attempts.

The structure of the paper is as follows. In “Methods” section we introduce and describe the experimental setting, we describe the collected data, and we define the metrics used to characterize the matches; in “Results” section we present the results of the data analysis; in “Discussion” section we comment on the outcomes and possible extensions of the work.

## Methods

### The Kreyon city experiment

**Figure 1:**
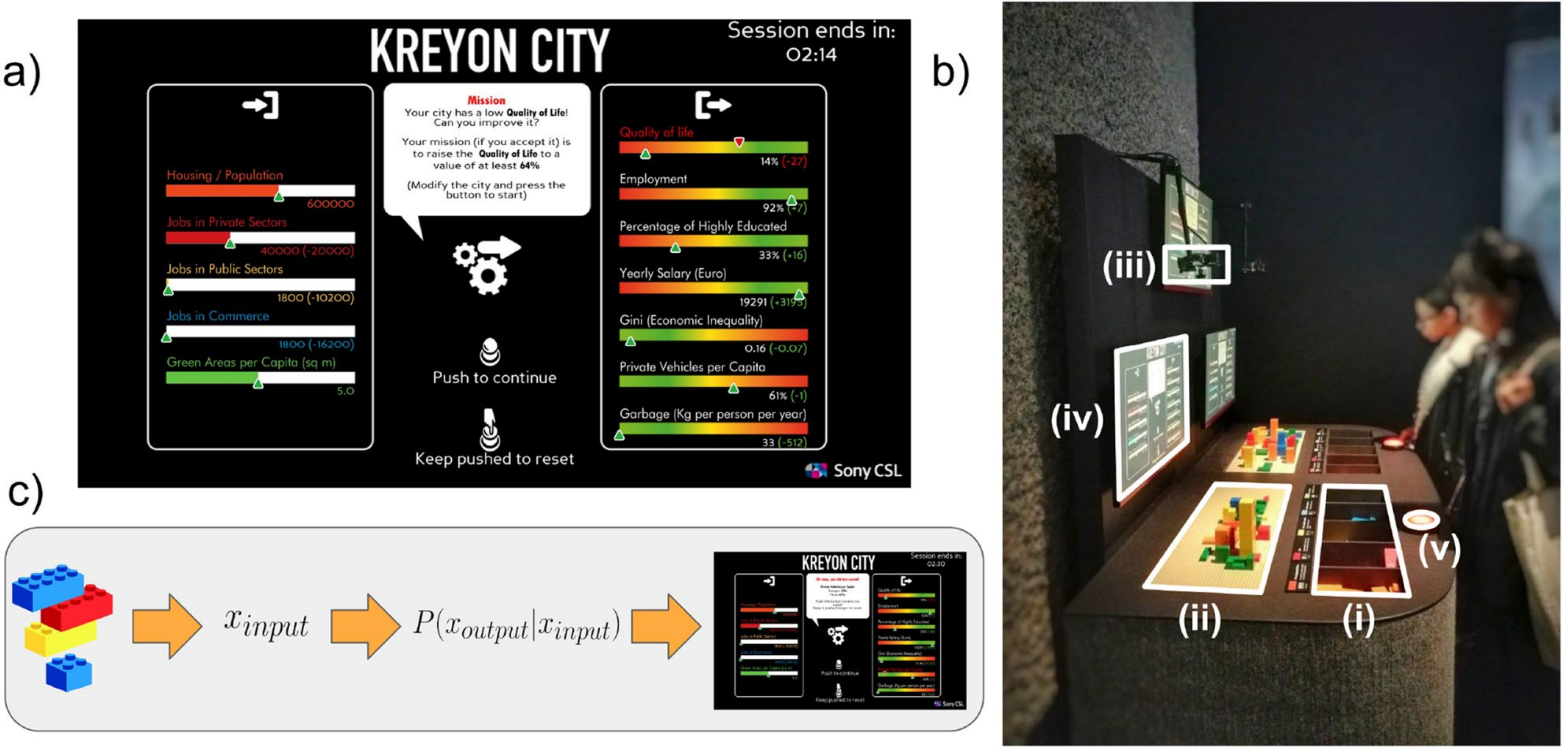
(**a**) Example of the interface. The mathematical model’s input is based on the number of bricks for each colour present in the building area. This input is displayed in the horizontal bars on the left part. The more the bricks of a specific colour, the more the bar would be filled. The outputs used to generate missions are shown in the right part of the interface. A green placeholder indicated their current value. Whenever one of the indicators became the target of a mission, a red placeholder appeared to guide the player towards the objective. (**b**) The installation with two gaming stations. Each gaming station had five containers to store bricks (i), a building area (ii) with an RGB/depth-sensor (iii) above it, a feedback monitor (iv) with a red button to control it (v). Brick containers (i) had labels attached on top providing a brief explanation of the brick colour meaning (e.g., “Orange bricks represents the population of the city”). (**c**) Scheme of user interaction during a mission. Once the player has modified the city and pressed the button, the RGB/dept-sensor translates the bricks’ number into the generative model’s input. The output is then computed as the most likely value of the probability distribution of the outputs conditioned on the input. The input corresponding to the different brick colours and the outputs are then updated simultaneously on the feedback monitor.

The experiment took place during the *AI: More than Human* exhibition held at the Barbican Centre in London (https://www.barbican.org.uk/whats-on/2019/event/ai-more-than-human) for around three months, from the $16{\textit{th}}$ of May to the $26{\textit{th}}$ of August 2019. During the whole duration of the exhibition, Kreyon City was on display, and visitors were free to access the installation at any time and take part in the experiment. The explanatory panel close to it presented the installation as an *interactive and playful experience that challenged participants to build their city while finding solutions for its problems*. The installation is shown in Fig. 1b. It consisted of a large table with two single-payer gaming stations. Each gaming station was composed of (i) 5 brick containers storing orange, red, yellow, blue, and green bricks; (ii) a building area with a base over which bricks could be attached; (iii) an RGB/depth-sensor placed on top of the building area; (iv) a feedback monitor placed in front of the player and (v) a button to control the game flow. The two gaming stations were controlled by one PC each, which managed both the sensors and the feedback monitors. The RGB/depth-sensor was calibrated to estimate the amounts of bricks of different colours on each building area.

The installation allowed for two people to play two separate and independent matches simultaneously. If no-one were playing on one station, the corresponding feedback monitor would show an introduction screen inviting visitors to play. The experiment started whenever an individual pushed the control button, hence triggering the second game screen. Each time the player pressed the button, a new screenshot was displayed, which was the only control for the installation’s digital part. After few steps with basic instructions and an explanation of the interface, the system proposed a mission to the player (see Fig. 1a for an example) and 5 min to accomplish it. The player then had to modify the city, adding or removing bricks of different colours. After each modification, the player was asked to push the button so to trigger the depth sensor. The sensor recorded the number of bricks of different colours on the board in the new configuration and sent the measurement to the computer that translated it into visual feedback via the underlying mathematical model. Through the feedback, the player knew whether she/he accomplished the mission. If not, the system prompted her/him to make another attempt. After five attempts, a final screenshot commented on the mission’s accomplishment and invited the participant to play again. At this point, a new match with a new mission was generated, unless the 5 min time already elapsed. In this case, the game was reset. If the installation was idle for more than two minutes, it reset automatically to the initial screenshot. A schematic and a more detailed representation of the game flow is shown in Section S1 of SI.

The measurements of the RGB/depth-sensors were translated into the input of a generative model to provide visual feedback. The model was trained using real census data (the collected data comes from the Italian Census, https://www.istat.it/it/archivio/104317, and the Urban Index website, https://www.urbanindex.it), following a Maximum Entropy inference approach^4^. To build the model, we considered a set of socio-economic indicators for each city $\alpha$, $\{X_i^\alpha \}_{i=1}^N$ and the city population $p^\alpha$. Note that we will drop the index $\alpha$ indicating the variable $X_i$ for a generic city $\alpha$. The set of indicators included: the number of jobs in the private sector, the number of jobs in the public administration and public services, number of people working in the commerce/retail sector, the surface of green areas within the city, quality of life indicator, the employment rate, the percentage of individuals with at least a Master Degree or have finished University, the average yearly salary, city’s Gini coefficient, the number of private vehicles per capita, amount of garbage produced per capita. It is well-known that these kinds of indicators follow a power-law relation with the city population^22,23^. Indicating with $a_i$ the exponent for corresponding power-law of each $X_i$, we can obtain the corresponding rescaled indicator as $x_i = \log _{10} \left( X_i/(X_i^0 p^{a_i})) \right)$. The model reproduces their multivariate probability of the rescaled indicators $\{x_i\}_{i=1}^N$ using the functional form

1 $$\begin{aligned} P(x_1,\dots , x_N) \propto \exp \left( -\sum _{ij} J^{(2)}_{ij}x_i x_j - \sum _{ijk} J^{(3)}_{ijk}x_i x_j x_k + \sum _{i} J^{(1)}_{i} x_i \right) , \end{aligned}$$

where the parameters $J^{(1)}$, $J^{(2)}$ and $J^{(3)}$ are inferred from the data (see Section 3 of SI for more details about the derivation of the model). It has been proven that how sampling from such model reproduces realistic configurations of urban indicators and that the presence of the $J^{(3)}$ term indicates the presence of effective non-linear interactions between them^4^. We divided the *N* indicators $X_i$ in two sets, $X_{\textit{input}}$ that could be controlled by the players together with the city’s population *p*, and $X_{\textit{output}}$ that were used as feedback about the city’s performances. To obtain $X_{\textit{output}}$ from $X_{\textit{input}}$ and *p*, we first rescaled the $X_{\textit{input}}$ using their power-law relation with *p* and obtaining $x_{\textit{input}}$. Then we used the conditional probability $P(x_{\textit{output}} | x_{\textit{input}})$ obtained from Eq. (1), that provides the most likely set of output values $x_{\textit{output}}$ in their rescaled version. Finally, we inverted the scaling relation for the $x_{\textit{output}}$ to obtain the output socio-economic indicators $X_{\textit{output}}$ in their standard form. The city population and the $X_{\textit{input}}$ indicators controlled by the player was represented with different colours:

- *Orange Bricks*: number of people living in the city.
- *Red Bricks*: number of people working in the private sector.
- *Yellow Bricks*: number of people working in the public administration and public services.
- *Blue Bricks*: number of people working in the commerce/retail sector.
- *Green Bricks*: the surface of green areas.

For readability’s sake, we will consider the population as part of the $X_{\textit{input}}$ vector from now on. We chose these input variables to be easily understandable for the average participant, yet to be able to represent the status of a city and to interact non-linearly with one another^4^. Each participant used the bricks to make buildings of different colours so that they represented different functional blocks of the city: e.g., orange buildings represented housing, thus influencing the total population; red buildings represented workplaces, affecting the number of jobs. The left part of the feedback monitor displayed these quantities as horizontal bars (Fig. 1a), that increase or decrease in size as the corresponding amount of bricks on the building area varies. The right part displayed the outputs $x_{\textit{output}}$ (Fig. 1a). Each output could be the target of a different mission, being each mission to increase or decrease its value within five attempts. They corresponded to:

- *Quality of Life*, an abstract indicator ranging from $0$ to $100\%$ indicating the level of life quality in the city. This is a compund indicator obtained from several indicators related to infrastructures, pollution, cultural life, wealth, employment, and public order;
- *Employment*, the percentage of the active population that has a job;
- *Percentage of Highly-Educated People*, the fraction of the population with a Master Degree or that finished University;
- *Yearly Salary*, average yearly salary in Euro;
- *Gini (Economic Inequality)* indicator quantifying the level of economic inequality;
- *Number of Private Vehicles per Capita*, the ratio between the number of private cars in the city and its inhabitants;
- *Garbage* average amount of garbage produced by an individual in a year, expressed in Kilograms.

Missions were generated by randomly choosing the worst-performing output indicator. The player was then asked to attempt to change that indicator’s value, moving it above or below a certain threshold. The threshold was chosen as larger than the current value for the beneficial indicators (the first four ones of the previous list) and a smaller value for the deprivation indexes (the last three of the list). Again, for the game to be intelligible for the general public, we provided only an overly simplified explanation about the target indicator and the task to be performed. To provide a rationale at the beginning of each mission, we displayed a brief text stating that a particular indicator’s specific values had adverse effects on the city. For example, whenever the garbage indicator was selected, the user would read a message stating: “*Citizens produce too much garbage! Can you improve the situation? Your mission (if you accept it) is to lower the garbage per capita and per year to a value of at least ...*”.

### Ethical approval and informed consent

Players were informed about the installation scientific nature and the game data collection before the experience. They agreed to data collection during the experiment (Figure S1 of SI). The experiment has been approved by the Ethics Committee of the KREYON Project, funded by the John Templeton Foundation under contract n. 51663, and by the Ethics Committee of Sony CSL Paris.

### Data collection and player identification

We collected data about player actions in the background, without interfering with the game experience. Whenever a player pushed the button in Fig. 1b, we created a timestamped record indicating at what point of the game the event occurred, together with the text displayed on the feedback monitor. We also recorded the number of bricks on the building area when the feedback asked to modify the city (see Section S2 of SI for more details of the types of records). As we did not log personal information, single records cannot be assigned directly to the different players. Still, it is possible to infer that the players had changed by looking at when the installation went idle and reset, and when the 5 min ended. In this way, we obtained several games made by a single player, each composed of several *matches*, i.e., the number of missions attempted. Since each player could keep playing as long as she/he wished, we decided to assign consecutive games to the same player if the time between the end of the 5 min (or the game’s voluntary reset) and the next button press was less than five seconds (see Section S2 of SI). Having identified the different players and their matches, we observed a general probability of 0.258 to successfully complete a match. However, that same probability drops to 0.06 when considering only the last match played by a player. This behaviour is consistent with the idea that many players could have left the installation at some point without resetting it. We then decided to exclude each player’s last observed match from the analysis, keeping it only if it was also the first. At the end of this process, we have a dataset of 8934 different matches. In Fig. S2 of SI, we show that the vast majority of players played only one match, but a fair number of them played two or three matches.

### Matches characterization

The collected data allows for the study of the players’ games at different levels. The units of analysis can range from the single moves performed by the players, to the players’ matches, to the players themselves. Here we are interested in identifying successful matches, so we introduce a set of metrics to characterize them and help understand the mechanism behind players’ ability to solve them.

#### Complexity of the missions

**Figure 2:**
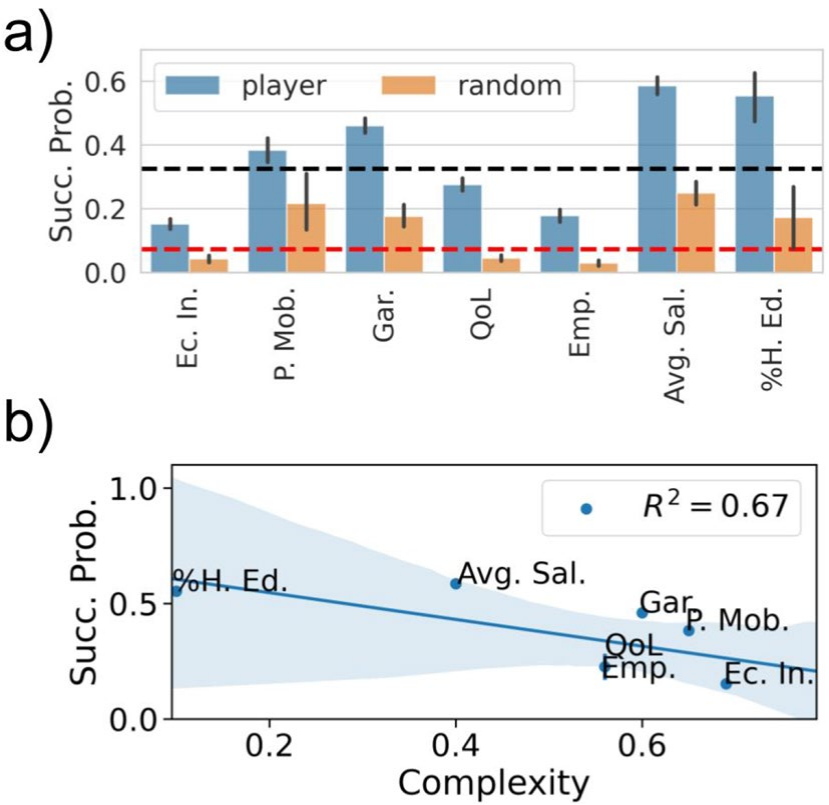
(**a**) Probability of success for the different mission types. Mission types are sorted in descending order according to their average complexity. Blue bars represent the results obtained with real players data, while orange bars represent those of the random agent. The black horizontal dashed line is the overall probability of success for real players, while the red one is for the random agent. Error bars have been computed as the standard error for a probability *f*, defined as $\sqrt{f(1-f)/n}$ where *n* is the size of the sample. (**b**) Correlation between the chances of accomplishing a specific mission type and its average Complexity. The solid line represents a linear regression between the two variables with the corresponding $R^2$ reported in the legend. The shaded area represents the 5–95% confidence interval. The mission labels in both panels correspond to the missions: Increase Quality of Life (QoL); Increase Employment Rate (Emp.); Increase Percentage of Highly-Educated individuals (%H. Ed.); Increase the Average Salary (Avg. Sal.); Decrease Economic Inequality (Ec. In.); Decrease the Number of Private Cars (P. Mob.); Decrease the produced Garbage (Gar.).

First, we defined a metric quantifying the intrinsic difficulty of accomplishing a specific mission. Figure 2a shows the probability of successfully completing each mission type, comparing it to the overall probability of success. Some missions are indeed more difficult, while others are accomplished one out of two tries. The missions players had to accomplish consisted of discovering, in the inputs’ space, a configuration $X_\textit{input}$ that corresponds, in agreement with the mathematical model, to a $X_\textit{output}$ that satisfies the requirements of the mission. Then the intrinsic **Complexity** of a mission is essentially the volume of the $X_\textit{input}$ space where the previous condition applies. Considering all the matches played by all the players, we defined a bounding box in the $X_\textit{input}$ space containing all the explored configurations. We discretized this bounding box creating a grid with $D=N^5$ points. For each point $X_\textit{input}^{(i)}$ with $i \in [1, D]$, we used our generative model to compute the corresponding outputs $X_\textit{output}^{(i)}$. As the model described by the probability (1) has been inferred using actual census data, players could impose $X_\textit{input}$ values beyond the limits observed in the data. In these areas, the model (1) gives information about the city by analytical continuation. For each match, we defined the **Complexity** of the assigned mission as 1 minus the fraction of grid points satisfying the mission conditions. With this definition, a match with Complexity equal to 0 if any of the configurations in the bounding box would solve the match. On the contrary, a Complexity equal to 1 indicates a match that cannot be completed in any way since there are no configurations in the bounding box that satisfy the mission’s conditions. Note that due to the experimental setup, we cannot control the initial configuration of each match. Players may start either from the configuration of their previous match or to the last player’s one. Thus, Complexity represents how hard it is to find configuration satisfying the match target, independently from where the player begins. We report a more detailed description in Section S4 of the SI.

#### Average move time and duration

Using the timestamps of player moves, we computed the **Average Move Time** as the average time elapsed two moves where a player has modified the city. We defined the match **Duration** as the time elapsed between the moments when the instructions about the mission were displayed and the display of the mission’s outcomes on the screen.

#### Non-linearity

The mathematical model driving the experience includes explicitly non-linear relations among all the input and output variables^4^. However, this non-linearity is not uniformly distributed on the input space so that players could experience it at different levels of intensity. Indeed, depending on the area of the configuration space they were playing in, some players could have experienced a completely linear response from the system. In contrast, others could have experienced fully non-linear effects. To quantify these different experiences, we compared the $x_\textit{output}$ of the different player’s moves with the output $x_\textit{output}^{\textit{L}}$ obtained with an entirely linear model (see Section S3 of SI for details about the derivation of this model). This model has a probability distribution that is similar to Eq. (1), but with only $J^{(2)}_{ij}$ parameters different from 0. Moreover, its $x_\textit{output}^{\textit{L}}$ can be explicitly computed using the equation:

2 $$\begin{aligned} x^{k}_\textit{output} = -\sum _{j \in \textit{output}} (J^{(2)})^{-1}_{kj} \sum _{i \in \textit{input}} J^{(2)}_{ji}x^{i}_\textit{input}. \end{aligned}$$

In the above equation, the *i* runs only over the input variable indices, *j* over the input and output variable indices, and *k* only on the output variable indices. **Non-linearity** of a configuration $x_\textit{input}$ is defined as the Euclidean distance $d(x_\textit{output}^L, x_\textit{output})$ between the linear output of Eq. (2) and the output $x_\textit{output}$ provided to the player during the game. In the following, we will focus on the **Initial Non-linearity** and the **Variation of Non-linearity**, i.e., the Non-linearity of the city configuration at the beginning of the match and the difference between the Non-linearity of the city configuration after the last player’s move and the beginning one. While the first metric characterizes the model non-linearity experienced by the player at the beginning of the match, the latter quantifies whether the player moved towards configurations associated with more or less non-linearity.

#### Paradox moves

To explore the game’s initially unknown space, players had to exploit their priors concerning the underlying model’s behaviour. Indeed, players could have built on their previous experiences to infer the system’s response to their moves. Then, some of these expectations could have not matched during the game. For example, it could be a common idea that increasing green areas should improve the Quality of Life. Still, in many cases, the model could predict that this is irrelevant or even detrimental, given the increased public expenses to maintain green areas. Since the current experimental setting did not allow for surveying players’ ideas before taking part in the experiment, we did two different surveys involving a panel of eight complex systems experts and a sample of 76 individuals living in the United Kingdom. While the participants to the first survey were voluntary, in the second case, the sample was recruited using MonkeySurvey (https://www.surveymonkey.com/). Neither the experts nor the non-experts took part directly in the experiment, but they were only asked to evaluate whether the increase or decrease of a specific socio-economic indicator (one of the game’s inputs) could solve a city’s issue (one of the game’s targets). As an example, we asked *Do you think that increasing the Population of the city would decrease the number of private cars?* to understand if, in their opinion, increasing the population indicator (orange bricks) could help to solve the mission-related with the Number Private Cars per Capita indicator. For each question, respondents had to assign a number equal to 1 if, in their opinion, increasing the input’s value could help to complete the output-related mission, − 1 if decreasing it would work, and 0 if, in their opinion, varying it was irrelevant. We computed the mean and standard deviations of each input-target combination’s answers, keeping separate the experts and non-experts groups’ answers. We defined consensus for a combination of whether the mean of the answers was different from 0 and larger than the standard deviation in absolute value.

Table S1 of SI shows the mean and standard deviations for the expert-survey answers. We can see that, for example, all the expert respondents agreed that increasing the number of people working in the private sector (red bricks) would help to accomplish the *Yearly Salary* mission. The consensus among the non-experts was not so strong, still in agreement with the latter (Table S2 of SI).

Considering one of those two tables, a **Paradox Move** is a move performed by a player that goes against the consensus (if reached by the respondents). In the previous example, paradox moves are all moves with a substantial decrease in the private sector (red bricks) when playing the *Yearly Salary* mission. Since our unit of analysis is the individual matches rather than single moves, we assigned a dummy value of 1 to all the matches in which at least one paradox move was identified.

See the Section S4 of SI for more precise definitions of these metrics. In the main text, we chose the experts’ survey results, but using the non-expert one led to similar results (see SI Section S6).

### Baseline random model

To assess whether players’ performances were compatible with random actions, we need to define a baseline random model. To do so, we first computed the distribution $P_i(\delta X_\textit{input}^{(i)})$ of the input variations $\delta X_\textit{input}^{(i)}$ made by the players (including the city’s population variations $P(\delta p)$). Then, we defined a random agent that played the randomly sampling the variations in each input from the distribution $P\left( \delta X_\textit{input}^{(1)} \dots \delta X_\textit{input }^{(N), delta p} \right) = \prod _i P_i \left( \delta X_\textit{input}^{(i)} \right) P(\delta p)$. In other words, the random agent performed the same player moves, but decorelating the variations for each input and disregarding the city configuration. We made the random agent play $10^4$ matches, collecting the same data we would collect for a real player. Every 10 matches, we reset the beginning configuration of the random agent with a random beginning configuration of a real match sampled from the data. In Table 1 we checked that the ranges of value explored for each $X_\textit{input}^{(i)}$ were consistent between the random agent and real players.

**Table 1:** Minimum and maximum observed values for the different input variables $x_\textit{input}^{(i)}$, made by the real players and the random agent.

	Population	Jobs Pri.	Jobs P.A.	Jobs comm.	Green area
Real players	$10^4$–$3.6\times 10^6$	1 − $2.74\times 10^6$	1 − $6.12^5$	1 − $1.144^5$	1 − $5.61\times 10^1$
Random agent	$10^4$–$3.32\times 10^6$	1 − $3.14\times 10^6$	1 − $6.35\times 10^5$	1 − $1.89\times 10^5$	1 − $1.61\times 10^1$

## Results

### Identifying successful matches

**Figure 3:**
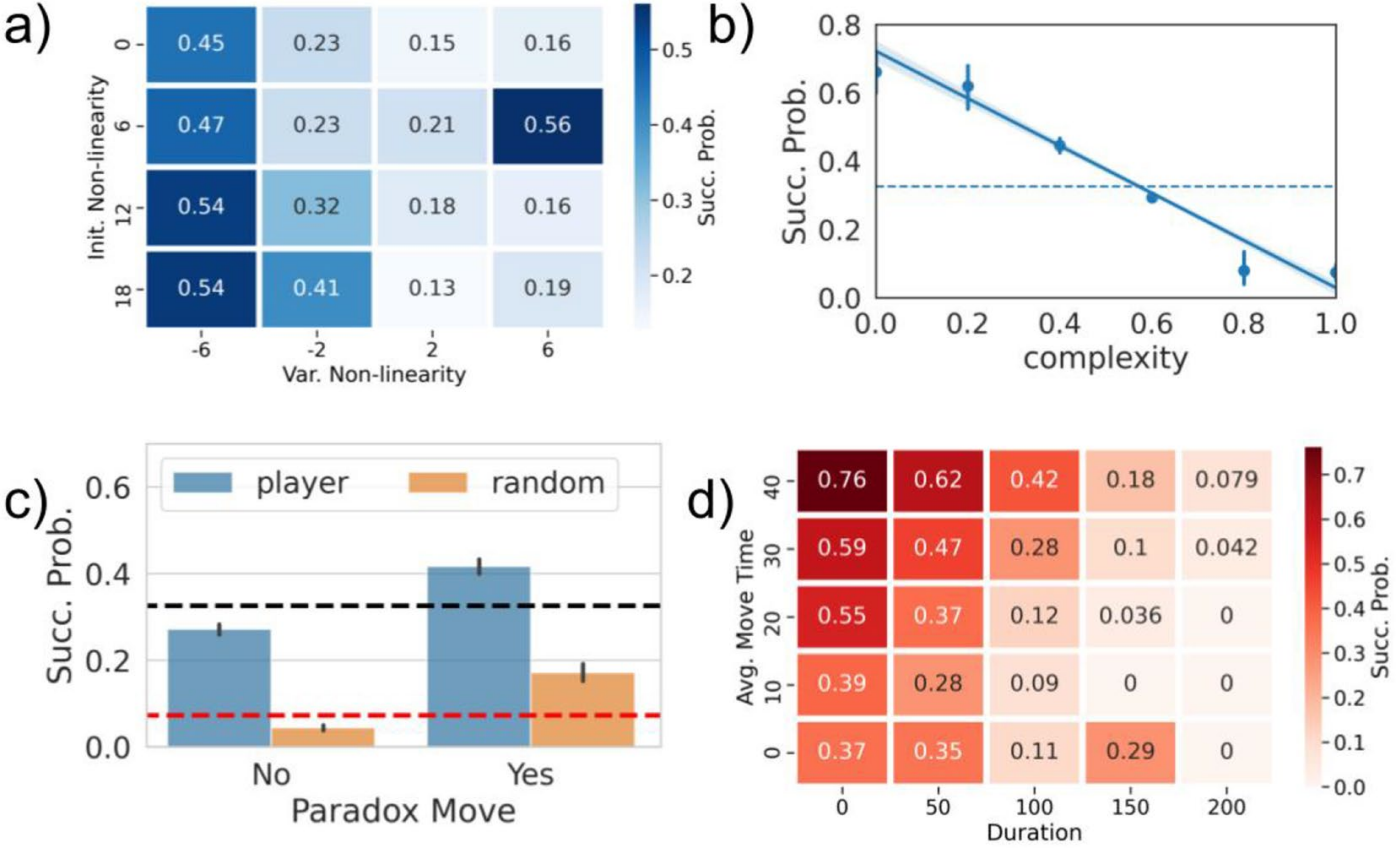
(**a**) Success probability as a function of Initial Non-Linearity and Variation of Non-Linearity. Success probability of a match vs. match complexity (**b**) and the presence of a paradox move (**c**). In (**c**), blue bars represent the results obtained with real players data, while orange bars result from the random agent. The horizontal dashed line (blue in (**b**) and black in (**c**)) is the overall probability of success for real players. The red horizontal dashed line in (**c**) is the probability of success for the random agent. Error bars have been computed as the standard error for a probability *f*, defined as $\sqrt{f(1-f)/n}$ where *n* is the size of the sample. The Paradox Move feature is defined using the experts’ survey results. (**d**) Success probability as a function of Duration and Average Move Time. Both these quantities are measured in seconds.

Figure 2a shows the success probability of all the players for the different mission types, as well as the same probability for the random agent. We can see that in all cases, real players are the best performers. Considering the overall success probability, the real players’ one is around $0.326\pm 0.005$ (corresponding to the black dashed lines in Figs. 2a,  3b,c), while random agent’s success probability is $0.097\pm 0.003$ (the red dashed lines in the same figures). The players’ best performances suggest some strategy or some understanding about the game functioning. To analyze how different match features are related to the outcome, we checked how they correlate with the success probability. The **Initial Non-Linearity ** and the **Variation of Non-Linearity** are highly interrelated metrics. Hence, in Fig. 3a we show how different combinations of these metrics’ values relate to the success probability. From Fig. 3a we can see how the best possible condition for a successful match is to start in a highly non-linear area and then move towards a more linear one, as the highest probabilities of success are clustered in the lower right part of the plot.

Figure 2b shows the anti-correlation between the average Complexity of all the matches with the same mission type and the success probability, proving that Complexity is a good proxy for the difficulty of a match. Figure 3b shows that this anti-correlation is even more evident without aggregating over the mission type, i.e., by measuring complexity for every single mission assigned to the players. As expected, matches with high complexity ($>0.75$) have rarely been accomplished by players since their solution space was too small to be found. Finally, Fig. 3c shows that the success probability for matches where at least a **paradox move** has been observed and for those where it was not. Matches where players have not made this kind of moves have a considerably lower success probability. On the other hand, paradox moves slightly enhance the success probability as compared to the average case. Moreover, in Fig. S5 of SI we show that in successful matches, paradox moves are typically performed at the end of the match, being thus likely to be decisive for the solution of the mission. In Section S7 of SI we show two examples of matches in which paradox moves have helped solving the mission and were the last performed move. Figure 3c shows the same results also for the random agent. Since, by definition, the paradox moves are helpful to solve the mission, the presence of a paradox move in the match increases the probability of it being a successful one also for the random agent. However, this does not increase the success probability enough to be comparable to the real players’ case. Moreover, the rate at which paradox moves can be found in a match is lower for the random agent: they can be found in around $23\%$ of random agent’s matches against and in $38\%$ of the real player ones. The **Duration ** and the **Average Move Time** are again highly interrelated since the time spent performing a move will undoubtedly impact the total duration of a match. In Fig. 3a show how combinations of these metrics are related with success. Success is indeed more likely in short matches (small duration) but high average move time. The best compromise seems to be when the player makes a few well-considered moves. This scenario is confirmed by noticing that the highest probabilities of success are observed for durations smaller than $100\;\text {s}$ with an Average Move time between 30 and $50\;\text {s}$.

**Figure 4:**
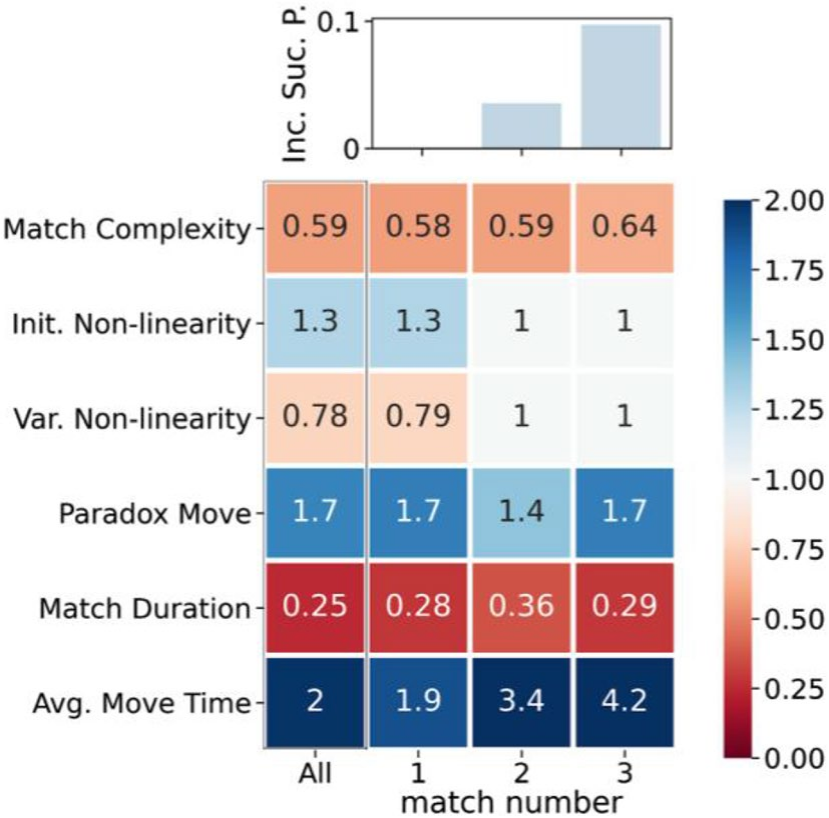
(Top) Increase of the success probability as a function of the match number. (Bottom) Odds ratios of the different features for predicting a successful match, for all matches together (first column) and each specific match number (other columns). An odds ratio equal to 1 indicates that the feature is irrelevant for the classification. We set to 1 all the odds ratios we found to be statistically indistinguishable to 1, using a *p*-value with a threshold of 0.05. An odds ratio larger than 1 indicates a positive correlation between the feature and a successful outcome. In other words, the feature’s presence raises the success probability. Conversely, an odds ratio smaller than 1 indicates a negative correlation, i.e., the feature’s presence reduces the success probability. The Paradox Move feature is defined using the experts’ survey results. Figure S5 in SI shows the same results for the non-experts case.

We checked this last result by performing a classification task using a simple Logistic Regression. Among several classification algorithms (e.g., the Random Forest Algorithm^24^), we selected the Logistic Regression for the highest readability of the results. The aim here is to discriminate between successful and unsuccessful matches. In our classification task, we assigned the label 1 to won matches, 0 otherwise and we train the Logistic Regressor to discriminate between these two cases. The input features used to train the classifier are the Match Complexity (continuous variable ranging from 0 to 1); the Avg. Move Time and Match Duration (continuous variables in seconds); the Initial Non-Linearity and Variation in Non-Linearity (both continuous variables; Paradox Move (dummy variable equal to 0 or 1). We have controlled for multi-collinearity issues between the features by computing the Variance Inflation Factor (VIF) for each of them, checking that it is always smaller than 2. We also split the sample of all the matches in a train and test set with a proportion of 70–30%, using the train set to infer the parameters of the Logistic Regressor and the test set to compute accuracy metrics. We repeated this procedure for 100 randomized train and test sets, retraining the classifier each time. We obtained an average Area Under the ROC curve (AuROC) of 0.83, a classification accuracy of 0.77, and an $F_1$-score of 0.61. Figure 4 shows the odd ratios for this logistic regression in the first column of the table. Odds ratios represent the constant effect of each feature on the match outcome. A feature with an odd ratio equal to 1 indicates that the feature is irrelevant for the prediction. An odd larger than 1 indicates that as the feature value increases, so does the success probability (vice-versa if it is smaller than 1). We see that Initial Non-Linearity, the presence of a paradox move, and the Average Move Time have odds larger than 1, indicating that increasing their value would also increase the probability of a successful match. Complexity, Variation of Non-Linearity, and Duration have odds lower than 1 (in the matrix shown in Fig. 4), indicating that large values for these variables are related to unsuccessful matches. These results are consistent with the previous analysis performed over a single variable or a couple of variables. Due to the experiment’s very nature and its particular setting, many individuals played only one match (see Section S2 of SI). However, we have a significant amount of players that iterated the game up to three times. In Fig. 4 (top panel), we show the increase in the success probability on the second and third match with respect to the first one. We see an increase of less than $5\%$ on the second match and around $10\%$ on the third one. Hence, players who iterated the game could be more effective and accomplish the mission more easily as they got more experienced. Thus, we can wonder whether anything changed in their perception of the game itself or whether they developed particular strategies. To partially answer this question, we repeated the classification task by dividing the data according to the match number. In general, we find a good precision for all the classification tasks, with AuROC larger than 0.82, $F_1$-score larger than 0.5, and accuracy larger than 0.76 in all cases. Since our sample is unbalanced towards lost matches, we also compared these accuracy metrics with those of a dummy classifier always predicting a loss. We found that the performances of the dummy classifier are considerably lower than those of the Logistic Regression (see Section S5 of SI for details). In Fig. 4 (bottom panel), we report the odds ratios for the classification tasks for different iterations of the game. While the odds ratios for the first played match are the same as the overall case, the situation changes in further iterations. We find an increase in the odds ratios of the Average Move Time for the second and third matches, indicating that it has become a more important feature. On the contrary, Initial Non-Linearity and Variation of Non-Linearity tend to become irrelevant with the number of matches. Given that the classification accuracy is still pretty high for these matches, we argue that the game’s non-linearity has already become evident to the player so that being in linear or non-linear parts of the $x_{\textit{input}}$ space is not relevant anymore. In contrast, being able to perform paradox moves is always relevant at any stage of the game.

## Discussion

Understanding how individuals relate to sustainability is becoming more critical due to the increasing complexity of our urban environments and public opinion’s impact on decision making and regulations. This work presented a novel, open-ended experiment that engages individuals in solving urban sustainability problems playfully, allowing them to interact with a complex generative model trained on real urban data. Each player had to search for specific urban configurations in terms of jobs, population, and green areas satisfying sustainability conditions such as the employment rate or the level of garbage produced. A fictional city made of LEGO bricks represented the configuration space to explore. Participants could modify the city and then check their modifications’ outcomes regarding urban socio-economic indicators on a screen. The experiment took place during an AI-devoted exhibition, and it allowed us to continuously gather data about the individuals’ interactions with the game for several months.

We were able to identify the determinants that could make a player successful in her/his interaction, i.e., succeeding in completing the installation’s missions. We showed that the non-linearity of the generative model is among these determinants. However, we showed that, through subsequent matches, players improve their understanding of the model’s underlying non-linearity and the interconnections between the city’s different components.

The other identified determinants were (1) the intrinsic complexity of the task, quantified using the size of the space satisfying the sustainability conditions; (2) the ability to perform moves challenging common beliefs about a specific issue; (3) the ability to perform few well-thought-out moves instead of several fast moves; While players overcame non-linearity, these other determinants remained relevant determinants of a successful match at every iteration. We compared some results with those obtained with a random agent, playing without considering the city configuration and the relations between input variables. We showed that, despite having explored similar configuration spaces, real players had better performances and could perform paradox moves more frequently. While all these results suggest a certain level of understanding of the game by participants, we would like to remark here that our results must be viewed only as circumstantial evidence of players’ learning process. We should provide further evidence to strengthen these results by extending the experimental setup to ask the participants why they perform particular moves, investigating whether they were the result of random attempts, understanding of the game mechanisms, or some prior knowledge about urban sustainability. We could also test the effect of participants’ notions about urban sustainability by repeating the experiment on a control group without labelling the input and output variables and just telling the participants that there are hidden relations between the bars in the feedback monitor. The experimental setup easily allows for these further iterations that we leave for future works. The proposed framework allows monitoring people during their learning phase for a challenging problem, like a sustainability problem. The framework is readily applicable to other kinds of complex problems. Modifying the underling model to encode spatial interactions between urban elements would allow to study issues like urban sprawl^25^ and public transportation^26^ and how to foster public understanding of it. The current generative model is general enough to include all sorts of urban socio-economic indicators. E.g. adding environmental-related variables would allow studying pollution issues^27^. The experimental setup is readily reproducible in other settings, either public venues or in controlled experiments. The current setup does not allow to control or stratify the experiment’s participants. Hence, our results might be affected by self-selection bias^28,29^ (e.g. participants are selected only among people attending art exhibitions, and only those interested in interactive games or urban sustainability could have taken part). Replicating the experiment in a controlled setting could help to understand whether our results can be generalized to the whole population.

The generative model itself could have given another bias. We trained it on data from a specific country that is not the one in which the experiment was carried. This might have hindered the participants understanding of the urban problems. However, modifying the generative model to reproduce different data is straightforward and could be implemented in future venues. In the current format, the experiment allows for collecting data in different countries or demographically different communities (e.g., suburban or urban, socially disadvantaged, etc.) would also allow for studying cultural differences in approaching sustainability problems and different national opinions about a city’s functioning. The opinions and strategies collected with the Kreyon City experiment could be valuable assets for decision-makers when confronting public opinion preferences and expectations. In fact, the game can be rearranged to propose only specific issues of interest to the participants, and help gathering data about their understanding of the problem and the solutions they found. Even more, Kreyon City can be itself a tool to pave the way to broader and more conscious participation of the population in the formulation of proposals and the decision-making process. Though the experiment was conceived for the study of individual behaviour, a different configuration and use of tools for monitoring the social interactions^20^ among participants will allow for studies of collective problem-solving. In this arrangement, it will be possible to connect the exchange of information with the game performances, thereby giving information on the social interactions that can lead to better and more diverse solutions to sustainability challenges. Besides, this type of multi-player configuration could be used as a boundary object in contested or conflictual situations to facilitate more open and productive dialogues^30–32^.

## Supplementary Information

Supplementary Information.

## Data Availability

The datasets generated during and/or analysed during the current study are available from the corresponding author on reasonable request.
