# Supplementary material for: Finding successful strategies in a complex urban sustainability game
Source: Sci Rep. 2021 Aug 3;11:15765. doi: 10.1038/s41598-021-95199-w (PMC8333319; doi:10.1038/s41598-021-95199-w)
Supplement: Supplementary file 1 — Supplementary Information. [file 41598_2021_95199_MOESM1_ESM.pdf]

Supporting Information for: Finding Successful Strategies in a Complex Urban Sustainability Game

Bernardo Monechi[1], Enrico Ubaldi[1], Pietro Gravino[1], Ilan Chabay[2], and Vittorio Loreto[1,3,4]

[1]Sony Computer Science Laboratories, 6, Rue Amyot, 75005, Paris, France
[2]Institute for Advanced Sustainability Studies, Berliner Strasse 130, 14467 Potsdam, Germany
[3]Sapienza University of Rome, Physics Department, Piazzale Aldo Moro 2, 00185, Rome, Italy
[4]Complexity Science Hub Vienna, Josefstädter Strasse 39, A-1080 Vienna, Austria

S1 Game Mechanics

Fig. S1 shows the game mechanics of the Kreyon City installation, showing the different screens displayed on the feedback monitors at each step. When a player approaches the installation, the feedback monitor displays the screen (i) in Fig. S1, with a generic message describing the installation and inviting the player to press the red button on the table (as shown in Fig. 1 of the main text); if the button is pressed the display goes to the screen (ii) where the player is informed about the scientific nature of the installation, and the fact that game data will be collected but sensitive data will not; if the player presses the button again, he/she agrees to play, and a screen (iii) showing the inputs and outputs of the model is displayed, stating that the inputs correspond to the bricks on the building area; pressing the button again leads to screen (iv) in which a mission is proposed to the player, who is invited to modify the city and press the red button again. From now onwards, a five minutes timer appears in the top right corner; the player then has five attempts to perform a "modify the city and press the button" action (v). After each button press, the outputs are computed using the number of bricks on the table at the moment; after five attempts or if the five minutes have elapsed, the game stops (vi), and we display the outcome of the mission (success or failure). After this, if the available time is not over, another mission is proposed (screen (iv)), and the game starts again. Otherwise, the game ends, and the installation resets to screen (i). Note that players are not forced to leave the installation and could keep on playing after the time has expired. At any time, if more than two minutes have elapsed since the last time the red button on the table was pressed, the game resets to screen (i). The player can also reset the game by keeping pressed the red button for more than five seconds. This reset instruction is displayed at each step of the game.

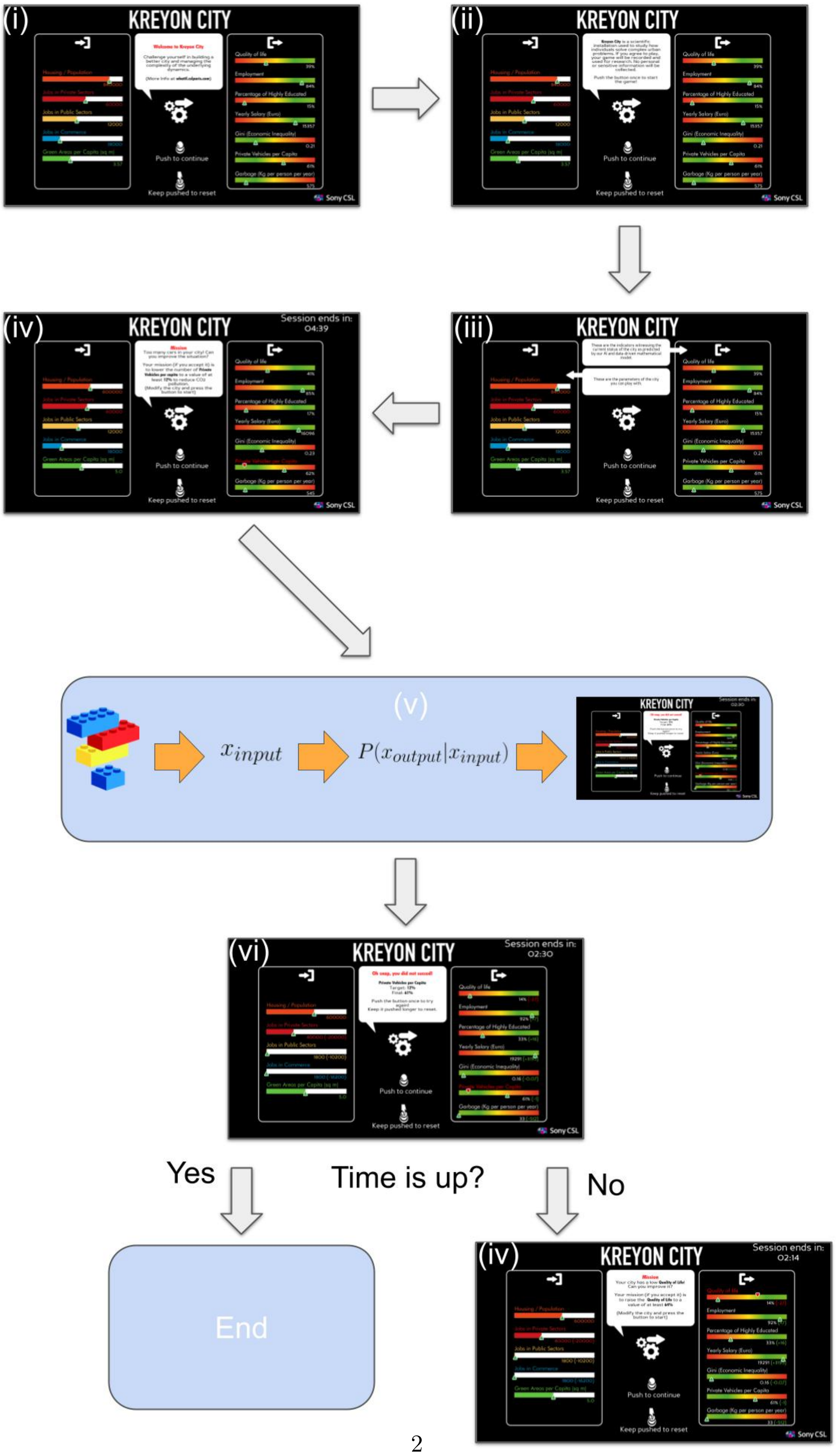

Figure S1: Schematic representation of the mechanics of the game.

S2 Description of the data

The data (number of bricks on the board, undergoing mission details and time) are automatically collected every time the player presses the red button or every time that the game automatically resets to the starting screen. The final dataset consists of a list of timestamped events, labeled according to the event-type. During some phases of the game, the record can also have information about the number of bricks on the board and the monitor's output values. The list of events is:

- **show_begin_screen**: generated whenever the begin screen (i) in Fig. S1 is displayed after button press or reset.
- **showUI_expl**: generated whenever screen (ii) in Fig. S1 is displayed after button press.
- **show_mission**: generated whenever screen (iii) with a mission is proposed to the player. Data about the number of bricks and the outputs variables' values are also collected and used as a starting point for the next mission.
- **attempt**: generated after button press during (iv). Data about the number of bricks and outputs are also collected. This data represents the move made by the player during a mission.
- **outcome**: generated whenever the outcome screen (v) is shown to the player. In this case, a flag variable is stored, being it equal to 1 if the mission is accomplished and 0 otherwise. A similar flag is also stored, marking whether the five minutes were over.
- **reset**: this record is generated whenever the game resets (if idle or after a long button press).

As we do not have direct information about the players, we need to identify them and associate them the specific matches. At first, we identified all the sequence of records starting from **show_begin_screen** and ending with **outcome** (with five minutes elapsed flag) or **reset**. These sequences represented a *game* made by a player, starting from the first button press. All **reset** events are considered as the game's ending with a loss. Since, in principle, a player is not forced to quit the installation after the end of the game, we defined a simple rule to assess whether she/he did not leave. Since screen (i) and (ii) are just initial instructions for the game, individuals having already played will likely skip them. Hence, if the time spent between a **reset** event or the last **outcome** event of a game, and the first **show_mission** event of the next one is less than five seconds, we considered the two games as the same and associated to the same player. Considering a *game*, different *matches* (i.e., attempts to accomplish a mission) are identified as all the events between each **show_mission** and the next **outcome** or **reset** events found. Each *match* will be characterized by a set of **attempt** events in which the player has modified the city. We first discarded all the *matches* in which no attempts have been recorded. As explained in the main text, we also discarded the last *match* played in each *games*, if there were more than one. Finally, we discarded all *games* without any valid *match*. The dataset provided with this work is the results of this data cleaning process and contains all the set of attempts performed by the players, together with information about the kind of mission played and wether the player won or not. At the end of this process, we collected 8934 matches played by 7585 different players. Fig. S2(a) shows the average number of players per hour for each building area of the installation. In both cases, we see that, on average, up to twelve players per hour were interacting with the installation. Fig. S2(b) shows the distribution of players having played at least one, two, three, and four matches. While the vast majority played only one match, 969 played up to two matches, 281 up to three, and 130 played more than three matches.

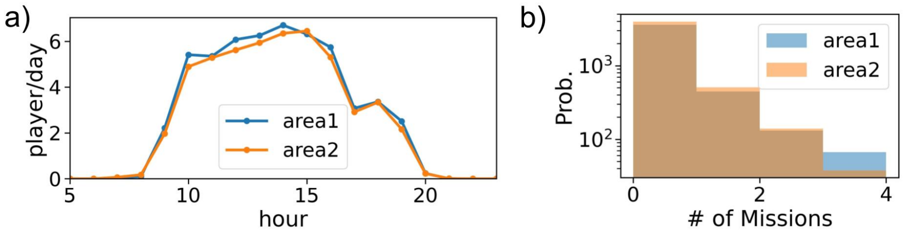

Figure S2: (a) The average number of players per area per hour in the two building areas of the installation. (b) The number of players who have played at least one, two, three, and four matches in the installation's two building-areas.

S3 Generative Model of Socio-Economic Indicators

We built the generative model used in this work following [1]. We first collected a set of indicators $X_i^{(\alpha)}$ where $i \in [1, N]$ is the index of the indicators and $\alpha \in [1, M]$ is the index of the city or town. We also collected the population P_α of each city/town. The data we collected come from the Italian census[1] and the Urban Index[2] website. The data cover all Italy's municipalities in 2011. The indicators collected are:

- The number of jobs in a city (excluding public services and retail);
- The number of jobs in public services;
- The number of jobs in commerce and retail;
- The extension per inhabitant of the green areas in the city;
- A compound quality of life indicator;
- The employment rate of the city;
- The percentage of inhabitants having attended to university;
- The average yearly salary in Euros;
- The Gini Coefficient of each city, calculated using the distribution of the salaries;
- The average number of private vehicles per inhabitant;
- The amount of garbage produced per inhabitant in a year.

Urban socio-economic indicators typically depend on the city's population in a power-law fashion [2, 3]. We can avoid explicitly using the population p_α in the modeling scheme due to this strong dependency. Indicating with a_i the exponent of the corresponding power-law relation between $X_i^{(\alpha)}$ and P_α, we then define the *rescaled indicators* as

$$x'^{(\alpha)}_i = \log_{10}(X_i^{(\alpha)}/(X_i^0 p_\alpha^{a_i})). \tag{S1}$$

[1] https://www.istat.it/it/archivio/104317
[2] https://www.urbanindex.it/

These indicators are, in principle, independent from p_α, so that we can avoid using the population directly in the generative model. We infer the model directly from the data, using the Maximum Entropy (ME) framework [4]. This framework's core is the definition of a set of observables $\langle O_\lambda \rangle_{data}$ defined on the data, that we consider as relevant for the description of the data. With this definition, the inferred probability distribution of the data $P_{ME}(x)$ is obtained through the maximization of the functional:

$$\Gamma[P_{ME}] = S[P_{ME}] + \sum_\lambda J_\lambda \left(\langle O_\lambda \rangle_{data} - \langle O_\lambda \rangle_{P_{ME}} \right), \tag{S2}$$

where $S[P_{ME}] = -\int dx P_{ME}(x) \log P_{ME}(x)$ is the Entropy of the distribution P_{ME} and $\langle f \rangle_{P_{ME}} = \int dx f(x) P_{ME}(x)$ is the average of the function f over the distribution P_{ME}. Thus, the maximization of equation (S2) is equivalent to maximize the Entropy of P_{ME}, subject to the constraints that the averages of the observables according to P_{ME} are equal to the experimental ones. With some straightforward calculation, we can find an explicit expression for P_{ME}:

$$P_{ME}(x) = \frac{1}{Z} \exp\left(-\sum_\lambda J_\lambda O_\lambda(x) \right), \tag{S3}$$

where the J_λ are the same Lagrange multipliers in (S2) and Z is the normalization constant of the distribution. The value of the multipliers can be inferred from the data, maximizing the Log-Likelihood:

$$\mathcal{L}(J_\lambda) = \frac{1}{N_c} \sum_\alpha \log P_{ME}(x^\alpha; J_\lambda). \tag{S4}$$

This Log-Likelihood can be maximized via gradient ascent provided that the gradients are known. The gradients can be expressed as:

$$\frac{\partial \mathcal{L}}{\partial J_\lambda} = \langle O_\lambda \rangle_{P_{ME}} - \langle O_\lambda \rangle_{data}. \tag{S5}$$

Thus, they are the difference between the average experimental observables used to define the model and the average obtained by sampling from P_{ME}. Despite that neither (S4) nor (S5) can be explicitly calculated, they can be approximated using numerical techniques [1]. Following [1], we find that the simplest choice for the $\langle O_\lambda \rangle_{data}$ are the correlations between indicators at several orders. In particular we will use the average of the indicators $C_i^{(1)} = \langle x_i \rangle_{data}$; the 2-points correlations $C_{i,j}^{(2)} = \langle x_i x_j \rangle_{data}$; and the 3-points correlations $C_{i,j,k}^{(3)} = \langle x_i x_j x_k \rangle_{data}$. Correlations of higher-order have a too large estimated error, that does not allow determining if the observables $C^{(k)}$ with $k > 3$ are different from 0. The final distribution that we obtain is

$$P_{ME}(x) \propto \exp\left(-\sum_{ij} J_{ij}^{(2)} x_i x_j - \sum_{ijk} J_{ijk}^{(3)} x_i x_j x_k + \sum_i J_i^{(1)} x_i \right), \tag{S6}$$

whose parameters $J^{(1)}$, $J^{(2)}$ and $J^{(3)}$ have been numerically inferred following [1]. It is not possible to sample directly from (S6) since the normalization constant Z cannot be explicitly calculated. Hence, we need to use numerical simulation to compute observables and expectation values. In the main text, we divided the variables in two groups X_{input} and X_{output}, including in the first the population p. While the first set of variables is controlled by the players, the second one has to be provided using the expectation value of $P(x_{output}|x_{input})$. Note that the population p is among the variables X_{input} but it is not directly part of (S6), which is defined on the rescaled indicators of equation (S1). The procedure to obtain X_{output} is then:

- Use Equation (S1) to obtain the rescaled version x^S_{input} of the inputs.
- Sample from $P_{ME}(x)(x^S_{output}|x^S_{input})$ using numerical methods (e.g. Metropolis-Hastings [5]). The non-normalized conditional probability $P_{ME}(x)(x^S_{output}|x^S_{input})$ can be easily obtained from (S6), by clamping the values of x^S_{input} and considering only the x^S_{output} as random variables. We then compute the average $\langle x^S_{output}\rangle_{P_{ME}}$ using the generated sample.
- We invert equation (S1) and we use it on $\langle x^S_{output}\rangle_{P_{ME}}$ to obtain the non-rescaled version of the output indicators X_{output}.

Note that the inclusion of $C^{(3)}_{i,j,k}$ in the set of relevant observable leads to a model that allows for **non-linear relations** between indicators [1]. To prove that the model can reproduce non-linear relations between indicators, we follow the results from the previous work [1], comparing 2-points, 3-points and 4-points correlations computed using the data and using a synthetic sample obtained from the probability distribution (S6). Fig. S3 shows the comparison between these correlations.

Figure S3: Comparisons between the correlators of order 2 (left), 3 (centre), and 4 (right) obtained with the empirical data (y-axis) and the by sampling from the model (S6)(x-axis). We report the percentage of components of each correlation which is not compatible with the data via a t-test with p-value 0.05 (For more details please refer to the original work [1]).

This, in turn, can lead to a complex landscape in the dependencies between x_{input} and x_{output}. Excluding this observable lead instead to a simpler and linear model, defined by the probability:

$$P^L_{ME}(x) \propto \exp\left(-\sum_{ij} J^{(2)}_{ij} x_i x_j\right). \tag{S7}$$

In this case we do not need to use numerical methods to compute x_{output} given x_{input}. In the rescaled indicator space, we find that for $k \in output$:

$$x^S_k = -\sum_{j\in output} (J^{(2)})^{-1}_{kj} \sum_{i\in input} J^{(2)}_{ji} x^S_i, \tag{S8}$$

that is x^S_k depends linearly on the x^S_{input}.

S4 Definitions of Match Features

In the main text, we defined five different metrics we used to characterize individual matches and to predict whether the match was successful or not. These metrics rely either on the raw data described in Section S2 or the generative model described in Section S3. In the **Paradox Move** metrics, we also surveyed a small sample of individuals to understand general opinions about the game's functioning.

S4.1 Match Duration

We compute this metric as the time elapsed between *show_mission* record and that of *outcome* record of each match (*reset* record if the game was reset).

S4.2 Average Move Time

We computed this metric as the average between the time elapsed between all the consecutive *attempt* records of the match. The time elapsed between the *show_mission* record and the first *attempt* record is also used.

S4.3 Match Complexity

This metric characterizes how many configurations of x_{input} satisfy the match's mission constraints. Since x_{input} is continuous and multidimensional and the generative model is analytically intractable, we need to use a discrete representation of the x_{input} space. To do so, we collect all the x_{input} value provided by the players in each match. Then we build a multidimensional bounding box using the maximum and minimum values observed for each input variable. We then divide each dimension into N equally spaced points to have a grid with $D = N^5$ points. We used a value of $N = 8$, but we observed that slightly larger or smaller values do not change the results shown in this work. Each point in the grid is then a x^i_{input} with $i \in [1, D]$ and we can follow Section S3 to obtain a corresponding x^i_{output}. All the game's missions are designed such that one of the variables in x_{output} is larger or smaller than a certain threshold. If we call the set of x^i_{output} satisfying this condition as S, the complexity of the mission is given by $C = 1 - \frac{S}{D}$. This quantity equals 0 if all the grid points satisfy the mission condition (low complexity) and 1 if none of them does (high complexity).

S4.4 Non-Linearity

This metrics characterizes how much the response of the generative model was non-linear after a player's attempt. In Section S3 we explain how it is possible to define a version of the generative model, where the relations between the socio-economic indicators are linear (equation (S8)). In general, the non-linearity of the generative model is not constant in the whole rescaled indicator space defined with (S1), and in some of its parts, it is almost equivalent to the linear model. To quantify the non-linearity of the response, we computed for each x_{input} of the *attempt* records of a match, the corresponding response of the linear model x^L_{output} using equation (S8). An attempt's Non-Linearity is then defined as the euclidean distance $d(x^L_{output}, x_{output})$ between this linear output and the output provided to the player. A value $d = 0$ indicates that the output was computed in a region of the x_{input} space where the model is completely linear. The larger the value, the highest the degree of Non-linearity the player has experienced. In the main text, we defined the **Initial Non-Linearity** metrics as the Non-Linearity value of configuration of the city at the beginning of the match, and the **Variation of Non-Linearity** metrics as the difference between the the configuration after the last attempt and the beginning configuration.

S4.5 Paradox Moves

This metric characterizes if a specific move challenges common beliefs about the functioning of the game. As explained in the main text, the generative model's behaviour can change according to the region of the x_{input} space the participants are playing in. Hence, it is essential to be flexible in making choices and performing counter-intuitive moves. To characterize what is considered *intuitive*, we performed two different surveys: one involving eight experts in Complex Systems, and the other involving 76 non-expert individuals living in the United Kingdom (the same nation were the

exhibition took place) recruited using the SurveyMonkey platform[3]. The non-experts were chosen to be the most representative possible of the United Kingdom population regarding age and gender. Fig. S4 shows the distribution of age and gender of the non-expert respondents. In both cases, we

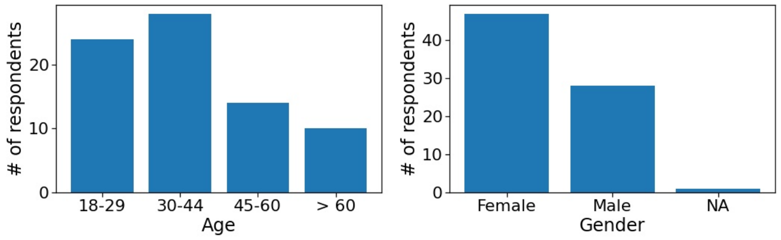

Figure S4: Distribution of the age (left) and the gender (right) of the respondents recruited using SurveyMonkey.

asked respondents to code whether changing a particular value of x_{intput} could help accomplish the mission. Before answering, we provided a brief explanation of the game mechanics as if they were regular players approaching the installation for the first time. Respondents had to answer a total of 35 questions, one for each combination of input and output variables. They had to assign a value of 1 if, in their opinion, increasing the input's value could help completing the output-related mission, -1 if decreasing it would work, and 0 if, in their opinion, varying it was irrelevant. The mean and standard error of respondents' answers are shown in Table S1 and Table S2 for the experts and non-experts cases, respectively. We consider equal to 0 all the mean values smaller than their standard error in absolute value. We can see that for some "input-output" combinations in the experts' case, the full consensus has been reached, i.e., all the respondents answered in the same way, and the mean value is exactly equal to 1 or -1 with the standard error equal to 0. Consensus for the non-experts surveys is generally weaker. However, the signed agreement with the experts' survey is on 23 questions over 35. In the main text, we use Table S1 as a reference for what a

Table S1: This table shows the mean and standard error of expert respondents' answers about the game mechanics. A positive value indicates that, on average, coders agreed that increasing the column's variable would help accomplishing the row's mission, a negative value that decreasing it would help. A value consistent with 0 indicates that changing the variable is irrelevant or that there is no clear consensus among the coders.

Mission	Population (orange)	Jobs in private sectors (red)	Jobs in P.A. (yellow)	Jobs in Commerce (blue)	Green Areas (green)
Incr. Quality of Life	-0.83 ± 0.15	1 ± 0	1 ± 0	0.66 ± 0.19	1 ± 0
Incr. Employment	-0.67 ± 0.30	1 ± 0	1 ± 0	1 ± 0	0.17 ± 0.15
Incr. % of Highly Educated	0.33 ± 0.19	0.83 ± 0.15	0.5 ± 0.2	-0.17 ± 0.15	0.17 ± 0.15
Incr. Yearly Salaries	-0.17 ± 0.28	1 ± 0	0.00 ± 0.33	0.33 ± 0.3	-0.17 ± 0.15
Decr. Economic Inequality	-0.5 ± 0.2	-0.5 ± 0.2	1 ± 0	-0.33 ± 0.3	0.17 ± 0.15
Decr. % of Private Vehicles	-0.67 ± 0.30	-0.83 ± 0.15	0.67 ± 0.19	-0.83 ± 0.15	0.50 ± 0.32
Decr. Produced Garbage	1 ± 0	-0.83 ± 0.15	0.67 ± 0.30	-1 ± 0	1 ± 0

[3]https://www.surveymonkey.com/

Table S2: This table shows the mean and standard error of non-expert respondents' answers about the game mechanics. A positive value indicates that, on average, coders agreed that increasing the column's variable would help accomplish the row's mission, a negative value that decreasing it would help. A value consistent with 0 indicates that changing the variable is irrelevant or that there is no clear consensus among the coders.

Mission	Population (orange)	Jobs in private sectors (red)	Jobs in P.A. (yellow)	Jobs in Commerce (blue)	Green Areas (green)
Incr. Quality of Life	-0.21 ± 0.10	0.24 ± 0.09	0.25 ± 0.08	0.03 ± 0.09	0.50 ± 0.09
Incr. Employment	-0.21 ± 0.09	0.28 ± 0.10	0.36 ± 0.09	0.33 ± 0.09	0.18 ± 0.08
Incr. % of Highly Educated	-0.30 ± 0.09	0.18 ± 0.08	0.13 ± 0.08	0.12 ± 0.08	0.11 ± 0.08
Incr. Yearly Salaries	-0.30 ± 0.09	0.01 ± 0.09	0.04 ± 0.08	0.08 ± 0.09	0.11 ± 0.07
Decr. Economic Inequality	-0.29 ± 0.09	0.11 ± 0.09	0.05 ± 0.09	-0.07 ± 0.09	0.04 ± 0.07
Decr. % of Private Vehicles	-0.19 ± 0.10	-0.18 ± 0.09	-0.33 ± 0.09	-0.25 ± 0.09	0.17 ± 0.08
Decr. Produced Garbage	-0.54 ± 0.09	-0.34 ± 0.09	-0.28 ± 0.09	-0.37 ± 0.09	0.09 ± 0.08

common belief about the functioning of the game might be before playing the game. We consider a player's modification of the city to be following this consensus if all the changes in the different colours are in the direction of the coders' consensus, not considering colours for which such consensus has not been observed. If at least one colour has changed in the opposite direction, we consider this a **Paradox Move**. To filter out fluctuations and unintentional Paradox Moves, we used a threshold on the colour variation. Considering a specific mission m, we first compute the average value $\bar{\delta}_{i,m}$ of the absolute value of the variation of the input variable i measured on all the matches referring to mission m played by all the players. Hence, we consider a move to be a Paradox Move only if the variation $\delta_{i,m}$ of at least one input variable i is: i) in the opposite direction of that predicted by Table S1 (or Table S2) for the mission m, and, ii) $|\delta_{i,m}| > \bar{\delta}_{i,m}$, i.e., the absolute input variable i variation is larger than the average color variation measured on all the other matches of mission m. For example, if a player trying to accomplish the *Increase Yearly Salaries* mission decreases the number of jobs in the private sector (red bricks) more than the average change in red bricks observed during the mission, this would be considered as Paradox Move. To assign a Paradox Move feature to a match, we assign 1 to each match with at least one Paradox Move among the player's attempts and 0 otherwise. The increase of the success probability shown in Fig. 4 of the main text suggests that Paradox Moves are connected to the strategies required to successfully complete a match. To provide more evidence about this connection, we can show that Paradox Moves typically occur at the end of the match, i.e., they are likely to be the move that allows accomplishing the mission. Given a match with a total number of attempts A, we can define, for each recorded move, the rescaled attempt number $r = a/A$, a being the attempt where a paradox move was recorded. This number is 1 if the attempt is the last performed in the match. Fig. S5 shows the average rescaled attempt number for Paradox Move and regular moves in the case of successful (panel a) and unsuccessful (panel b) matches. While there is no particular difference between the averages in the latter case, the average is higher in the case of successful matches. This fact indicates that, in successful matches, Paradox Moves have occurred, on average, toward the end of the match. Note that the results shown in Fig. 4 of the main text and in Fig. S5 are qualitatively the same if instead of Table S1, we would have used the non-experts one in Table S2 (not shown).

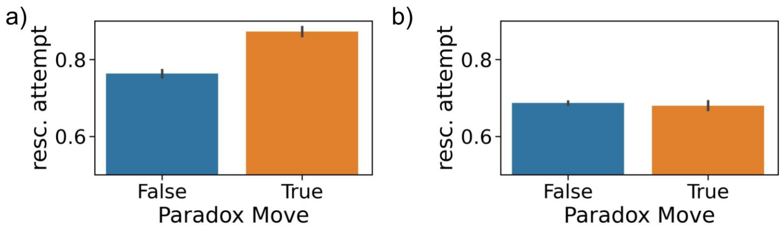

Figure S5: Average rescaled attempt number for Paradox Moves (orange) and regular moves (blue) for successful (a) and unsuccessful (b) matches.

S5 Match Classification Tasks

In the main text, we presented four different classification tasks performed on individual matches. Each task's goal is to classify matches according to their outcomes. We assigned a flag equal to 1 if the player accomplished the mission and 0 otherwise. We assigned to each match the set of features described in Section S4, and we used them as independent variables for a Logistic Regression Classifier. Logistic Regression is probably the simplest way to build a classifier, and it also features high readability. The different tasks correspond to the case in which all the matches are classified together or grouped by the match number (first, second, or third match). It is essential for each task to control for multicollinearity between independent variables since it could lead to unstable estimates of the model parameter. A simple way to do this is to compute the Variance Inflation Factor (VIF). A variable with a VIF larger than 5 indicates high collinearity between it and some other variables, so removing it should be considered. Table S3 shows the VIF for all the independent variables in all the classification tasks. No value of VIF larger than 2 has been observed. For each

Table S3: This table shows the Variable Inflation Factors for all the features (rows) used in the different classification tasks (columns). A value larger than 5 indicates high collinearity and potential errors in estimating the model's parameters.

Variable	All matches	First Match	Second Match	Third Match
Match Complexity	1.05	1.06	1.06	1.03
Init. Non-Linearity	1.42	1.42	1.49	1.51
Var. Non-Linearity	1.40	1.39	1.48	1.52
Paradox Move	1.06	1.14	1.18	1.14
Match Duration	1.64	1.25	1.16	1.17
Avg. Move Time	1.65	1.14	1.10	1.14

classification, we divided the sample of the matches into training and test sets so that the training set would be the 70% of the whole dataset. Then we used the training set to learn the model parameters and the test set to check the model's predictive power. We repeat this procedure 100 times for each classification, randomizing the training and test sets each time. Table S4 shows some classification precision metrics obtained by averaging over the 100 randomizations. In particular, we computed the Area Under the Receiver Operating Characteristic Curve (AuROC), the classification accuracy, and the F1-score. We see that good classification performance is achieved in all cases.

Table S4: This table shows the mean and standard error over the different randomizations of the classification accuracy metrics. The three bottom rows show the classification accuracy of dummy classifiers always predicting the most frequent value of the sample (loss). The Paradox Move feature is defined using expert respondents' answer (Table S1).

Variable	All matches	First Match	Second Match	Third Match
AuROC	0.8306 ± 0.0007	0.8210 ± 0.0007	0.838 ± 0.002	0.827 ± 0.003
Accuracy	0.7690 ± 0.0007	0.7756 ± 0.0007	0.775 ± 0.002	0.766 ± 0.003
F_1-score	0.601 ± 0.001	0.526 ± 0.001	0.81 ± 0.002	0.760 ± 0.003
AuROC (dummy)	0.5 ± 0.0	0.5 ± 0.0	0.5 ± 0.0	0.828 ± 0.003
Accuracy (dummy)	0.6654 ± 0.0007	0.7121 ± 0.0007	0.401 ± 0.002	0.437 ± 0.004
F_1-score (dummy)	0.0 ± 0.0	0.0 ± 0.0	0.0 ± 0.0	0.0 ± 0.0

S6 Match Classification Tasks (Non-experts survey)

In the main text and in Section S5 we used the Table S1, corresponding to experts respondents, to define Paradox Moves. This section shows that using the non-expert respondents' answers (Table S2) leads to similar results in terms of classification accuracy and features relevance, despite some disagreements with the expert respondents' answers. Table S5 shows the same classification precision metrics as Table S4, obtained by substituting the Paradox Move feature defined using Table S1, with the one obtained using Table S2. We show that in this case, too, there are good classification performances in all the tasks. Considering the odds of the features, we show them in Fig. S6 using the same format of Fig. 4 in the main text. The results for the two cases are qualitatively very similar.

Table S5: This table shows the mean and standard error over the different randomizations of the classification accuracy metrics. We do not report here the classification accuracy metrics since they are the same of Table S4).The Paradox Move feature is defined using non-experts respondents answers (Table S2).

Variable	All matches	First Match	Second Match	Third Match
AuROC	0.8399 ± 0.0007	0.8331 ± 0.0007	0.838 ± 0.002	0.828 ± 0.003
Accuracy	0.78021 ± 0.0007	0.7933 ± 0.0008	0.783 ± 0.002	0.760 ± 0.004
F_1-score	0.632 ± 0.001	0.5652 ± 0.002	0.82 ± 0.002	0.760 ± 0.003

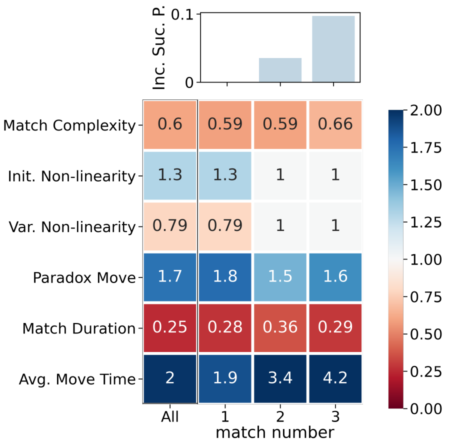

Figure S6: Same results as Fig. 4 in the main text, using the Paradox Move featured defined using the non-experts survey results. Also in this case we report the odds for the different features in all the classifications task of successful matches. An odd equal to 1 indicates that the feature is irrelevant for the classification. We set to 1 all the odds we found to be statistically indistinguishable to 1, using p-value with a threshold of 0.05.

S7 Example of Matches

In this section, we show two examples of games played by two different players. In both cases, Fig. S7 and Fig. S8, the players played an unsuccessful match and then another successful one that they solved with a paradox move. In the first case, Fig. S8 the player made initially several attempts to solve the same mission type until he found a solution by performing a paradox move, moving almost all the variables in the opposite direction with respect to those obtained from Table S1. The second example shows a similar case, but the player had to solve two different mission types. He solves the second one by decreasing the number of jobs in the commerce sector (blue bricks). This is in opposition to the experts' consensus from Table S1 concerning the *Increase Yearly Salary* mission.

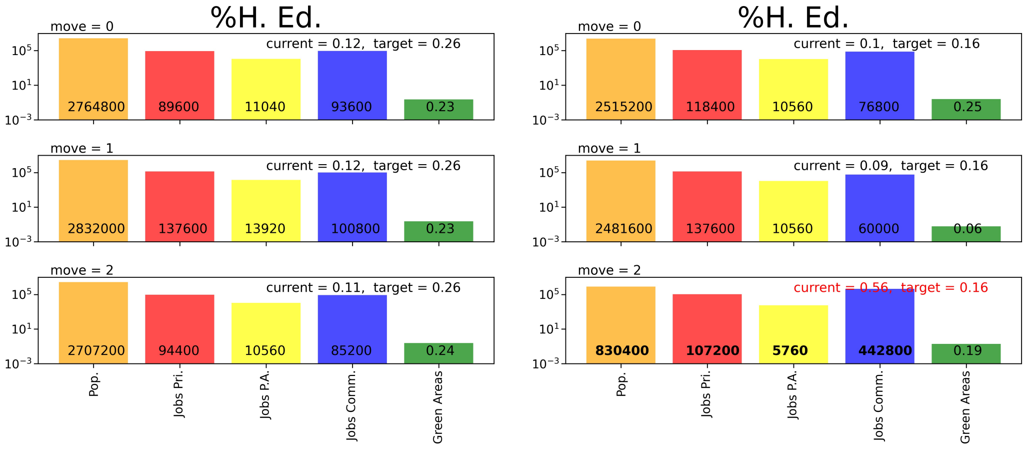

Figure S7: Example of two subsequent matches (the left one has been played for first) played by the same player. In both cases the player had to increase the *Percentage of Highly-Educated People* above 26% in the first match and above 56% in the second match. Vertical bars of different colours represent the input value after each player's move, while each panel represent a different move. The numeric value within each bar is the corresponding value of the variable. We report the numeric value of a variable in bold, if a paradox move was performed by varying it. The value of the target output variable after each move is shown in the legend.

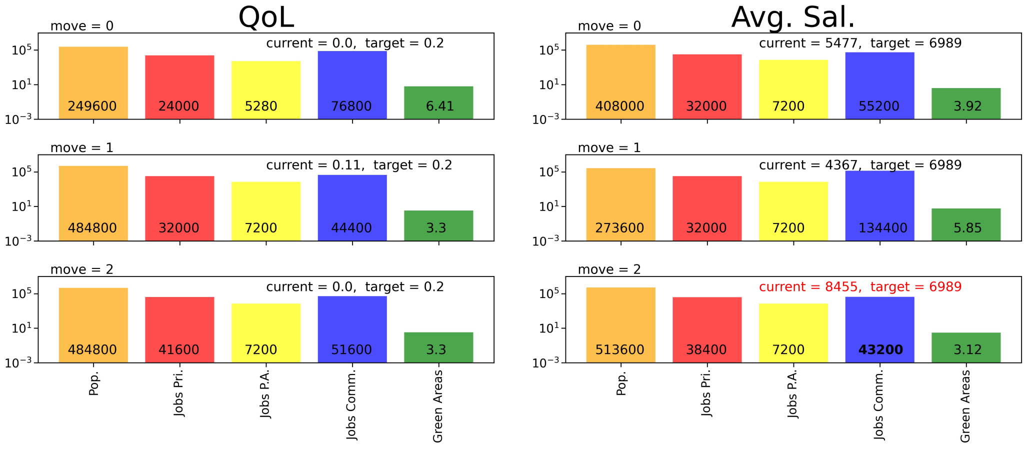

Figure S8: Example of two subsequent matches (the left one has been played for first) played by the same player. In the first match the player had to increase the *Quality of Life* above 20, and in the second the *Average Yearly Salary* above 8455. Vertical bars of different colours represent the input value after each player's move, while each panel represent a different move. The numeric value within each bar is the corresponding value of the variable. We report the numeric value of a variable in bold, if a paradox move was performed by varying it. The value of the target output variable after each move is shown in the legend.

References

[1] Monechi, B., Ibáñez-Berganza, M. & Loreto, V. Hamiltonian modelling of macro-economic urban dynamics. *R. Soc. open sci.* **7**, 7200667 (2020).

[2] Bettencourt, L. M., Lobo, J., Helbing, D., Kühnert, C. & West, G. B. Growth, innovation, scaling, and the pace of life in cities. *Proceedings of the national academy of sciences* **104**, 7301–7306 (2007).

[3] Bettencourt, L. M., Lobo, J., Strumsky, D. & West, G. B. Urban scaling and its deviations: Revealing the structure of wealth, innovation and crime across cities. *PloS one* **5**, e13541 (2010).

[4] Martyushev, L. M. & Seleznev, V. D. Maximum entropy production principle in physics, chemistry and biology. *Physics reports* **426**, 1–45 (2006).

[5] Robert, C. P. & Casella, G. The metropolis—hastings algorithm. In *Monte Carlo Statistical Methods*, 231–283 (Springer, 1999).
